# The frequency of *TP53* R72P and *MDM2* 309T>G polymorphisms in Iranian infertile men with spermatogenetic failure: A case-control study

**Published:** 2018-08

**Authors:** Zeinab Ebrahim Abadi, Maryam Khademi Bami, Maryam Golzadeh, Seyed Mehdi Kalantar, Mohammad Hasan Sheikhha

**Affiliations:** 1 *Department of Medical Genetics, Medical Faculty, Shahid Sadoughi University of Medical Sciences, Yazd, Iran.*; 2 *Medical Biotechnology Research Center, Ashkezar Branch, Islamic Azad University, Ashkezar, Iran.*; 3 *Abortion Research Center, Yazd Reproductive Sciences Institute, Shahid Sadoughi University of Medical Sciences, Yazd, Iran.*; 4 *Research and Clinical Center for Infertility, Yazd Reproductive Sciences Institute, Shahid Sadoughi University of Medical Sciences, Yazd, Iran.*; 5 *Biotechnology Research Center, International Campus, Shahid Sadoughi University of Medical Sciences, Yazd, Iran.*

**Keywords:** Apoptosis, Male infertility, MDM2, TP53, Polymorphisms

## Abstract

**Background:**

Tumor protein *p53* (*TP53*) is a tumor suppressor transcriptional regulator protein which plays a critical role in the spermatogenesis. One of the most important regulators of p53 is Murine double minute 2 (*MDM2*), which acts as a negative regulator of the p53 pathway. Based on the key role of p53 and *MDM2* in germ cell apoptosis, polymorphisms that cause a change in their function might affect germ cell apoptosis and the risk of male infertility.

**Objective:**

This study was designed to examine associations of *TP53* 72 Arg>Pro (rs1042522), and *MDM2* 309 T>G (rs937283) polymorphisms with spermatogenetic failure in Iranian population.

**Materials and Methods:**

A case-control study was conducted with 150 nonobstructive azoospermia or severe oligozoospermia and 150 fertile controls. The two polymorphisms, 72 Arg>Pro in *TP53* and 309 T>G in *MDM2*, were genotyped using PCR-RFLP and ARMS-PCR respectively.

**Results:**

Our analyses revealed that the allele and genotype frequencies of the *TP53* R72P polymorphism were not significantly different between the cases and controls (p=0.41, p=0.40 respectively). Also, no significant differences were found in the allelic (p=0.46) and genotypic (p=0.78) distribution of *MDM2* 309 T>G polymorphism between patients and controls.

**Conclusion:**

The results of this study indicate that polymorphisms of *TP53* and *MDM2* genes are unlikely to contribute to the pathogenesis of male infertility with spermatogenetic failure.

## Introduction

Infertility is a major health problem that affects approximately 10-15% of childbearing ages couples; among which about half of the cases are attributable to male factors (1, 2). Previous studies have revealed that spermatogenic failure is the most common form of male infertility, which is presented in the forms of non-obstructive azoospermia (NOA) or Oligozoospermia (1, 2). However, the cause of most cases remains unknown (1). Germ cell apoptosis is a key step during normal spermatogenesis and is believed to play an important role in controlling the number of germ cells and eliminating defective germ cells to produce functional spermatozoa (3, 4). For instance, the apoptotic Fas/Fasl pathway can regulate normal sperm production and control of semen quality in human (5). Analysis of the genes involved in the apoptosis process that occurs during spermatogenesis proved that *TP53* and *MDM2* genes may have an important role in spermatogenesis (6). 

The spontaneous germ cell apoptosis and germ cell quality control in spermatogenesis is TP53 mediated. It is observed that TP53 knock-out mice noticeably have less normal spermatozoa compared with TP53+/+ mice; in consequence their fertility rate reduced and have a testicular giant-cell degenerative syndrome (3). MDM2 is a key negative regulator of p53 protein that can inhibit TP53 cell cycle arrest and apoptosis (7, 8). It has been reported that MDM2-/- mice die in the early embryonic stage of their life, while simultaneous knock-out of TP53 in them leads to normal development; so it can be deduced that MDM2 is a key negative regulator of TP53 during development (9). 

Numerous attempts have been made to identify single nucleotide polymorphisms (SNPs) affecting male infertility, such as *TP53* and *MDM2*, but the conclusions of previous studies were inconsistent. Therefore more study is required in order to clarify their association (10-12). Researchers have reported numerous SNPs in the p53 pathway and some of them may change the function of the TP53 protein. For example one of the important polymorphisms of the *TP53* gene occurs at codon 72, which results in a change from arginine (Arg) to proline (Pro) (13, 14). It has also been reported that the GG type of *MDM2* SNP309 can increase the MDM2 level and leads to attenuation of TP53 function (15). There is a large volume of published studies describing the role of polymorphisms in the apoptotic disorders such as cancers; however, few studies examine their impact on male infertility (16-21).

The present study aimed to examine the association between *p53* and *MDM2* polymorphisms and the risk of male infertility with spermatogenetic failure in Iran. In this study, we compared the genotypes of two polymorphisms between non-obstructive azoospermic and oligozoospermic men and healthy controls to evaluate the risk factor of male infertility for the first time.

## Materials and methods

**Subjects**

All studied subjects were consecutively recruited from the Research and Clinical Center for Infertility, Yazd, Iran, including 150 infertile men with NOA or oligozoospermia and 150 fertile men. All patients and controls were ethnic Iranian. Infertile men were chosen from couples refering to the center for infertility treatment. All of the patients in the study showed either azoospermia (absence of sperm in the ejaculated semen) or oligozoospermia (sperm count <15×10^6^ ml^-1^). Patients with seminal tract obstruction were excluded from the study according to the urologist examination. In total, 150 infertile men (96 NOA and 54 oligozoospermia) aged from 19-50 yr were enrolled in this study. The control group consists of 150 normozoospermic men with female factor subfertility.

**Genotyping**

DNA was extracted from the peripheral blood using GeneAll genomic DNA isolation kit according to the manufacturer's protocol (GeneAll ExgeneTM Blood SV mini, Korea). Genotypes were analyzed using PCR-based methods as described below. Genotyping was performed without knowledge of subjects case/control status. The genotypes of *TP53* Arg72pro (rs1042522, G>C) were analyzed by restriction fragment length polymorphism RFLP-PCR method (22). The primers used were *TP53* F, 5’-AGCAGAGACCTGTGGGAA GCGA-3’ and *TP53* R, 5’-CAGGGCAACTGAC CGTGCAAGT-3’, which produce a 483-bp fragment containing the G/C site. 

Amplification was accomplished with a 20 µl reaction mixture containing 1 µl template DNA (100 ng/ µl), 1 µl of each primer (10 pmol/µl), 7 µl dH_2_O and 10 µl Taq 2x Master Mix red (Ampliqon). PCR profile consisted of an initial melting step of 10 min at 94^o^C, followed by 30 cycles of 45 sec at 94^o^C, 45 sec at 67^o^C, and 45 sec at 72^o^C, and a final elongation step of 7 min at 72^o^C. The 483-bp PCR products were then digested overnight by restriction enzyme *Bstu1* (New England Biolabs, UK). After digestion with *Bstu1*, PCR product was cut into 310-bp and 173-bp in the presence of G allele, whereas the C allele was undigested.

*MDM2* SNP309 (rs2279744, T>G) genotypes were analyzed using the tetra-primer amplification refractory mutation system (ARMS) PCR method (23). Primers *MDM2* F1, 5’- GGGGGCCGGGGGCTGCGG GGCCGTTT-3’ and *MDM2* R1, 5’- TGCCCAC TGAACCGGCCCAATCCCGCCCAG-3’ were used to amplify the 122-bp wild-type allele (T) and primers *MDM2* F2, 5’-GGCAGTCGCCG CCAGGGAGGAGGGCGG-3’ and *MDM2* R2, 5’- ACCTGCGATCATCCGGACCTCCCGCGC TGC-3’ were used to amplify the 158-bp mutant allele (G) as previously reported (24). 

The amplification was accomplished with a 20 µl reaction mixture containing 1µl template DNA (100 ng/µl), 0.125 µl of primers *MDM2* F1 and *MDM2* R1 (10 pmol/µl), 0.25 µl of primers *MDM2* F2 and *MDM2* R2 (10 pmol/µl), 8.25 µl dH_2_O and 10 µl Taq 2x Master Mix red (Ampliqon). The reaction was carried out with an initial melting step of 10 min at 94^o^C, followed by 35 cycles of 45 sec at 94^o^C, 45 sec at 70^o^C, and 45 sec at 72^o^C, and a final elongation step of 7 min at 72^o^C. 

The amplified DNA was visualized on an agarose gel containing DNA green viewer TM (Pars-Tous Biotechnology, Iran). The *MDM2* SNP 309 T allele generated a 122-bp band, and the G allele generated a 158-bp band. They had a common 224-bp band, which was amplified by primers *MDM2* F2 and *MDM2* R1. To validate the genotyping results, randomly selected PCR amplified DNA samples (n=1, for each genotype) were examined by direct DNA sequencing and the results were also consistent.

**Ethical consideration**

Each subject signed an informed consent to participate in our study and to donate a blood sample for genomic DNA extraction. The study was approved by the Ethics Review Board of Research and Clinical Center for Infertility, Yazd, Iran (IR.SSU.MEDICINE.REC. 1394.344).

**Statistical analysis**

All statistical analysis was performed using SPSS software (Statistical package for the social science, version 19.0, SPSS Inc, Chicago, Illinois, USA). Differences in genotypes distributions between cases and the controls were examined by Pearson Chi-square test. The association between the *TP53* and *MDM2* polymorphisms and risk of spermatogenic failure were estimated by ORs and their 95% confidence intervals (CIs) from logistic regression analyses. The results were considered statistically significant when p<0.05.

## Results

**Figure 1:**
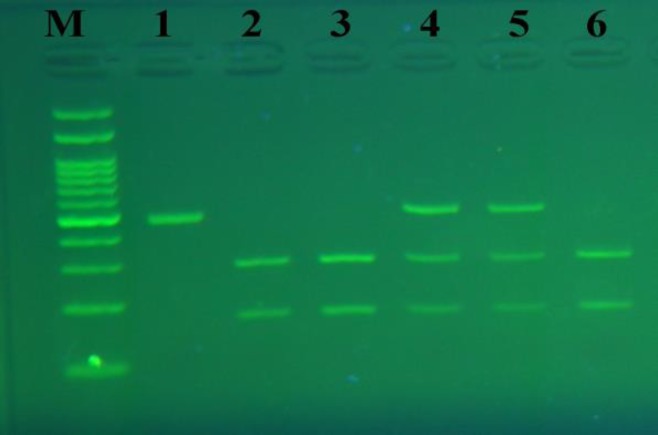
The results of polymerase chain reaction fragment length polymorphism (PCR-RFLP) analysis of the *p53 *R72P polymorphism. Lanes 1: undigested PCR product of 483 bp for Pro/Pro genotype, Lanes 2, 3, 6: two fragments of 310 and 173 bp for Arg/Arg genotype, Lanes 4, 5: three fragments of 310, 173 and 483 bp for Arg/Pro genotype. M: 100 bp DNA marker

**Figure 2:**
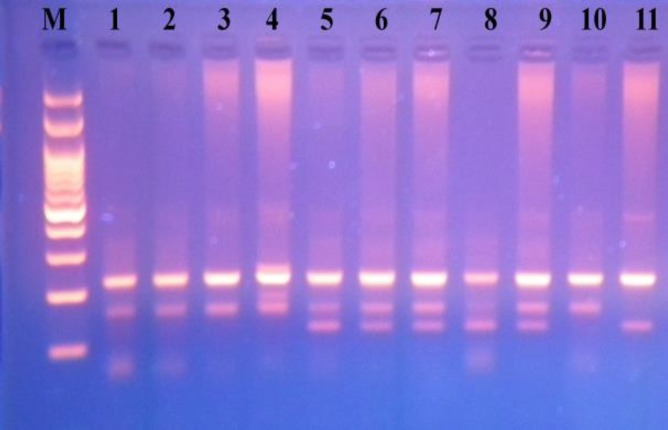
*MDM2 *SNP309 PCR product stained by Green viewer on 2% agarose gel electrophoresis. Lane 1, 2, 3, 4, 10: two fragments of 224 and 158 bp for GG genotype, Lane 5, 6, 7, 8, 9: three fragments of 158, 122 and 224 bp for TG genotype, Lane 11: two fragments of 224 and 122 bp for TT genotype. M: 100 bp DNA marker

This study included 150 patients with azoospermia or oligozoospermia (average age of 33.88 yr) and 150 healthy control subjects (average age of 33.58 yr). There were no statistically significant differences in the distribution of age between patients and controls (p>0.31). The observed genotype frequencies among the controls and cases were in concurrence with Hardy-Weinberg equilibrium. The PCR-RFLP results and ARMS-PCR results are shown in figure 1 and figure 2, respectively. The rs1042522 distribution and allele frequencies in 150 cases and 150 controls are displayed in table I. The analysis of rs937283 distribution and allele frequencies among groups and subgroups are presented in table II.

**Analysis of** ***p53*** **codon 72 polymorphism**

Our results indicated that the frequencies of GG, GC and CC genotypes in the infertile patients (30.7%, 52.7%, and 16.7% respectively) were similar to those in the controls (30%, 47.3%, and 22.7% respectively) (Table I). Using the CC genotypes as a reference, the OR of the GC genotype was 1.39 (95% CI=0.72-2.69) and the OR of the GG genotypes was 1.51 (95% CI=0.82-2.77). The statistical analysis showed that there was no differences in the allelic (p=0.41) and genotypic (p=0.40) distributions in the main groups as well as the sub group.

**Analysis of** ***MDM2*** **SNP 309T>G**

Our results showed that the frequencies of the GG, TG, and TT genotypes in the controls were, 30.3%, 42% and 26.7% respectively, which was simillar to these percentages in the infertile patients (28.7%, 41.3% and 30% respectively). Using the TT genotype as a reference, the OR of the TG genotype was 0.8 (95% CI=0.45-1.47) and the OR of the GG genotype was 0.87 (95% CI=0.5-1.52). Also, no significant differences were found in the allelic (p=0.46) and genotypic (p=0.78) distributions (Table II).

**Table I:** Genotype and allele frequencies of *TP53 *72 Arg>Pro (rs1042522) among the patients and controls and their associations with male infertility

**Genotype**	**Control**	**Case**	**Azoospermia**	**Oligospermia**
GG	45 (30)	46 (30.7)	25 (26)	21 (38.9)
CG	71 (47.3)	79 (52.7)	54 (56.3)	25 (46.3)
CC	34 (22.7)	25 (16.7)	17 (17.7)	8 (14.8)
p-value	-	0.40	0.38	0.34
Chi^2^	-	1.81	1.93	2.17
Allele*				
G	161 (53.7)	171 (57)	104 (54.2)	67 (62)
C	139 (46.3)	129 (43)	88 (45.8)	41 (38)
p-value	-	0.41	0.91	0.13
OR (95% CI)	-	1.14 (0.83-1.58)	1.02 (0.7-1.46)	1.14 (0.9-2.21)

Data presented as n (%). p<0.05 was considered statistically significant.

CI: Confidence interval

OR: Odds ratio

* Chi-squared test.

**Table II:** Genotype and allele frequencies of *MDM2 *5^' ^UTR T>G (rs937283) among the patients and controls and their associations with male infertility

**Genotype**	**Control**	**Case**	**Azoospermia**	**Oligospermia**
GG	47 (31.3)	43 (28.7)	29 (30.2)	14 (25.9)
TG	63 (42)	62 (41.3)	38 (39.6)	24 (44.4)
TT	40 (26.7)	45 (30)	29 (30.2)	16 (29.6)
p-value	-	0.78	0.83	0.75
Chi^2^	-	0.48	0.37	0.57
Allele*				
G	157 (52.3)	148 (49.3)	96 (50)	52 (48.1)
T	143 (47.7)	152 (50.7)	96 (50)	56 (51.9)
p-value	-	0.46	0.61	0.46
OR (95% CI)	-	1.06 (0.90-1.25)	0.91 (0.63-1.3)	0.85 (0.54-1.3)

Data presented as n (%). p<0.05 was considered statistically significant.

5' UTR: 5' untranslated region

CI: Confidence interval

OR: Odds ratio

* Chi-squared test.

## Discussion

The results of the present study showed that there are no significance differences between the studied SNPs pf *TP53* and *MDM2* genes and infertility in men.

Apoptosis is a key step that occurs during spermatogenesis to eliminate damaged germ cells in testis. A broad series of genes are functionally important in the apoptosis pathway (5). *TP53* and *MDM2* are both essential genes in the apoptosis process; these are thought to provide another level of stringency in addition to other spermatogenic quality control mechanism. It has been reported that if either the *TP53* or the *MDM2* pathway is functionally abnormal, for instance, some SNPs that affect their functions, infertility may occur. It was found that *TP53* 72 Arg>Pro and *MDM2* 309 T>G alters the activity or the level of the TP53 protein, and polymorphisms of the two genes are associated with the risk of apoptosis disorder diseases, such as various cancers (16-19). 

Based on the above information, we tested the hypothesis that the polymorphisms in the apoptosis pathway might be responsible for spermatogenetic failure and therefore the following male infertility. Based on the results of this study, there was no significant difference in allele and genotype distribution of the *TP53* and *MDM2* polymorphisms between cases and controls. This may indicate that these two polymorphisms are not contributed to the pathogenesis of spermatogenetic failure in male infertility. It remains possible that some other functional polymorphism in these genes might be candidates for evaluating individual susceptibility in male infertility. Several case-control studies have explored the association between the *p53* R72P polymorphism and male infertility, but the results are contradictory. 

Our results were in partial agreement with Huang and colleagues, Lu and colleagues and Ying and colleagues but different from Mashayekhi and Hadiyan and Jin and co-worker who suggested that Arg appears to increase the risk of developing idiopathic male infertility in Iranian and southeast Chinese men (9, 12, 25-27). Only one case-control study by Haung and colleagues has investigated the association of *MDM2* SNP 309 T>G with the risk of male infertility but our results do not support their research who reported a statistical association between *MDM2* SNP 309 T>G with idiopathic male infertility (25). Our results were consistent with previous data in cancer (28, 29).

Recently, Ying Chan and co-worker performed a meta-analysis based on the data from four currently available studies (9, 25-27) to clarify the association of *p53* R72P with male infertility. They also reported no association between rs1042522 and idiopathic male infertility (12).

Unlike the previous studies who investigated the frequency of *p53* R72P in cases and control groups, Jin's and our study analyzed the frequency of rs1042522 in NOA, oligozoospermia and control groups. The majority of our cases and Jin's cases were NOA which accounts for about 64% and 58.73% respectively. Considering subgroup analysis of Jin's study, they reported that rs1042522 was not relevant to the risk of spermatogenetic failure in oligozoospermia group (p=0.28), which was similar to our data in an Iranian population (p=0.34). They also found no statistically significant difference between allelic distribution among oligozoospermia compared with control (p=0.13) just like our's (p=0.13). It is possible that different subgroup population composition may have contributed to distinct results.

The results from our and Mashayekhi's research were not consistent with each other, although both involved an Iranian sample it indicates that the same ethnicity but different groups may also bring a divergent conclusion. There are several possible explanations for these contradict results. First is different inclusion criteria of studied population in our study with previous studies, as the cases in previous reports were idiopathic infertile men which means that cases were analyzed for a normal 46, XY karyotype and absence of Y chromosomal microdeletions of AZF region proved by molecular analysis. But our cases did not analyze for microdeletions of AZF region and normal karyotype, due to financial constraints. It was reported that Y-q microdeletion is accounted for a big proportion of infertility in men (30). The other factors include different sample size and composition, as the majority of our cases are NOA. 

Diverse ethnic backgrounds and effect of gene-environment interactions should also be taken into consideration. Genetic background and specific environment may interact with each other to affect fertility statue of individuals perhaps these two polymorphisms affect male fertility by combining with some other polymorphisms of genes or did not have inherited risk factor.

## Conclusion

This is the first study which aimed to investigate the association of *p53* R72P and *MDM2* SNP 309 T>G with the risk of male infertility in subgroup division of NOA and oligozoospermia in an Iranian population. In summary, we conclude that codon 72 polymorphism in *p53* gene and SNP 309 T>G in the *MDM2* gene were not relevant to spermatogenic failure in cases, NOA group and oligozoospermia group based on an Iranian population including 150 infertile men and 150 healthy controls.

Returning to the hypothesis posed at the beginning of this study, it is now possible to state that the examined polymorphisms in *p53* and *MDM2* might not be a good genetic marker for evaluating infertility in Iranian men. Further examination of a larger sample size is needed to confirm our results. Since male infertility is a heterogeneous disorder, with numerous environmental and genetic factors contributing to impaired spermatogenesis (31), more broadly research is also needed to determine environmental factors such as nutrition, smoking, and exercise.

## Note

***This article extracted from M.Sc. thesis. (Zeinab Ebrahim Abadi)***
